# Osmotic Stress Induced Cell Death in Wheat Is Alleviated by Tauroursodeoxycholic Acid and Involves Endoplasmic Reticulum Stress–Related Gene Expression

**DOI:** 10.3389/fpls.2017.00667

**Published:** 2017-05-03

**Authors:** Liting Zhang, Zeyu Xin, Xing Yu, Chao Ma, Weiwei Liang, Meichen Zhu, Qiwei Cheng, Zongzhen Li, Yanan Niu, Yongzhe Ren, Zhiqiang Wang, Tongbao Lin

**Affiliations:** ^1^College of Agronomy, Henan Agricultural UniversityZhengzhou, China; ^2^Collaborative Innovation Center of Henan Grain CropsZhengzhou, China; ^3^National Key Laboratory of Wheat and Maize Crop ScienceZhengzhou, China; ^4^College of Agronomy, Henan University of Science and TechnologyLuoyang, China

**Keywords:** osmotic stress, TUDCA, ER stress, ROS, cell death

## Abstract

Although, tauroursodeoxycholic acid (TUDCA) has been widely studied in mammalian cells because of its role in inhibiting apoptosis, its effects on plants remain almost unknown, especially in the case of crops such as wheat. In this study, we conducted a series of experiments to explore the effects and mechanisms of action of TUDCA on wheat growth and cell death induced by osmotic stress. Our results show that TUDCA: (1) ameliorates the impact of osmotic stress on wheat height, fresh weight, and water content; (2) alleviates the decrease in chlorophyll content as well as membrane damage caused by osmotic stress; (3) decreases the accumulation of reactive oxygen species (ROS) by increasing the activity of antioxidant enzymes under osmotic stress; and (4) to some extent alleviates osmotic stress–induced cell death probably by regulating endoplasmic reticulum (ER) stress–related gene expression, for example expression of the basic leucine zipper genes *bZIP60B* and *bZIP60D*, the binding proteins *BiP1* and *BiP2*, the protein disulfide isomerase *PDIL8-1*, and the glucose-regulated protein *GRP94*. We also propose a model that illustrates how TUDCA alleviates osmotic stress–related wheat cell death, which provides an important theoretical basis for improving plant stress adaptation and elucidates the mechanisms of ER stress–related plant osmotic stress resistance.

## Introduction

As one of the most important crop plants in the world, wheat plays an essential role in global food security. Generally grown in arid and semi-arid areas, wheat growth and development are greatly affected by drought, which is one of the main environmental factors limiting wheat production in China. In fact, studies have revealed that losses of wheat grain yield induced by drought are increasing year by year in most countries (Foulkes et al., 2007; Godfray et al., 2010; Li et al., 2011).

Plants develop numerous signal transduction pathways to respond to water stress (Griffiths, 2002; Umezawa et al., 2010; Kim et al., 2012; Todaka et al., 2015). An endoplasmic reticulum (ER) stress pathway has been reported to be involved in plant responses to environmental stresses (Valente et al., 2009; Liu and Howell, 2010a; Srivastava et al., 2014). However, the mechanism by which ER stress mediates plant drought tolerance is less known, especially in crop plants such as wheat.

The ER is an organelle in the cell where proteins are created and folded. Folding is a very elaborate process that is often interrupted by a variety of biotic and abiotic stresses, leading to the formation of unfolded and misfolded proteins. When the number of malformed proteins accumulates to levels that exceed the self-repairing capability of the ER, stress occurs as the ability of the cell to fix proteins is determined by ER quality control (ERQC) and ER-assisted degradation (ERAD) mechanisms (Liu and Howell, 2010a; Hüttner and Strasser, 2012; Cai et al., 2014; Cao and Kaufman, 2014). In mammals, ER stress signals are recognized by the signal sensors related to activating transcription factor 6 (ATF6), RNA-dependent protein kinase–like ER kinase (PERK), and inositol-requiring enzyme 1 (IRE1) (Liu and Howell, 2010a; Hetz, 2012; Williams et al., 2014), while in plants, ER stress is recognized by the major membrane-associated transcription factors (MTFs), along with bZIP17, bZIP28, and bZIP60 (Liu and Howell, 2010b; Sun et al., 2013; Yang et al., 2013; Srivastava et al., 2014). These ER stress signals subsequently activate an unfolded protein response (UPR) that reduces the accumulation of malformed proteins and alleviates the stress by positively regulating the expression of molecular chaperones (e.g., *BiP, PDI, CNX, CRT*) to promote protein folding, negatively regulating expression of secretory proteins to reduce protein production and load in the ER lumen, and increasing the degradation of malformed proteins via the ERAD pathway (Turano et al., 2002; Jia et al., 2008; Liu and Howell, 2010a; Han et al., 2011; Wang S. J. et al., 2012; Zhu et al., 2014). However, if severe conditions continue and ER stress cannot be alleviated, prolonged exposure leads to cell death and affects plant growth (Hetz, 2012; Sun et al., 2013; Cai et al., 2014; Yang et al., 2014). Although, there have been significant recent advances in research on the ER stress–mediated cell death pathway, large disparities remain compared with progress in research on mammals.

TUDCA is a chemical-chaperone that is often considered in ER stress–related studies on mammals as well as in clinical trials. This compound shares similar functions with other molecular chaperones in that it not only has the ability to stabilize misfolded proteins and polypeptides, but also plays an important role in protein transport and degradation (Phillips et al., 2008; Drack et al., 2012). Indeed, because of the ability of TUDCA to promote protein folding and relieve ER stress, this compound has the potential to be developed into a new kind of anti-stress agent in the future. Exploring the effects of TUDCA on plants under multiple stresses is therefore of great significance. Recent research has shown that TUDCA can be used to treat a number of protein-misfolding diseases and that it exhibits anti-apoptotic properties in mammals (Schoemaker et al., 2004; Schulz et al., 2009; Vandewynckel et al., 2015). In higher plants, studies have shown that this compound alleviates ER stress and cell death in *Arabidopsis thaliana* caused by the ER stress–inducers tunicamycin and dithiothreitol (Watanabe and Lam, 2008; Sun et al., 2013). Additional studies have also shown that TUDCA acts to inhibit heat- and Cd^2+^-induced ER stresses as well as cell apoptosis and augments heavy metal and heat stress tolerance in plants (Williams et al., 2010; Xu et al., 2013; Yang et al., 2014).

Although, a number of studies have addressed the effects of TUDCA on alleviating ER stress, there remains little research into the mechanisms by which this compound regulates osmotic stress–related ER stress and cell death. It remains unclear whether, or not, TUDCA can reduce cell death and mitigate the effects of osmotic stress on wheat. In addition, the mechanisms underlying this regulation are unknown. To address these questions, we conducted a series of studies to investigate plant responses to osmotic stress after TUDCA treatment using wheat seedlings, and researched the expression patterns of genes involved in the signal transduction pathway related to ER stress.

## Materials and methods

### Plant materials and treatments

We used the wheat cultivar YN211 in this study, which is one of the main wheat varieties grown in Henan Province, China. Surface-sterilized YN211 seeds were soaked in water for 12 h, and incubated on wire netting floating in water at 25°C in the dark for 3 days to allow germination. Seedlings were then removed from the wire netting, placed in half-strength Hoagland's nutrient solution (changed once every 3 days) in a phytotron at 25°C/22°C, and subjected to a photoperiod of 16 h of light and 8 h of darkness.

Based on our previous data, which revealed that applying 100 μg/mL TUDCA (Sigma-Aldrich, USA) had no obvious effect on seedling growth except for slightly improving the physiological characteristics of wheat leaves and enhancing the expression of *bZIP60* under normal growing conditions (Figures S1–S3), we used three experimental treatments in this study: control treatment, osmotic stress (polyethylene glycol, PEG-6000) treatment, and osmotic stress combined with pre-treatment of TUDCA (PEG + TUDCA). The last of these treatments involved spraying the foliage of seedlings with 100 μg/mL of TUDCA solution for 4 days when the plants were at the one-leaf stage. Osmotic stress, simulated by 20% PEG-6000 (−0.975 MPa), was then initiated following TUDCA pre-treatment before the plants were harvested and measured.

The wheat seedlings were allowed to grow for 15 days in a hydroponic system and samples were collected at different time points to detect the time course changes of various physiological and biochemical parameters (Figures S4–S6).

The experiments were replicated five times to ensure repeatability. We collected samples from the second replicated experiment onward. In each collection of four batches, three or four biological replicates were sampled and assayed for each morphological, physiological, biochemical, and molecular biological parameter.

### Growth parameters

We recorded a series of growth parameters after 4 days of PEG stress. Biomass weight was recorded after drying at 105°C for 30 min and at 80°C for 12 h, while plant height and root length were measured using a metric ruler. Absolute water content (AWC) was calculated using the following formula: AWC (%) = (fresh weight (FW) − dry weight (DW))/ FW × 100.

### Chlorophyll content

We measured chlorophyll content using ultraviolet (UV) spectrophotometry according to the method described by Sartory and Grobbelaar (1984) with minor modifications. To do this, the latest fully expanded leaf of each wheat seeding was collected, cut into 5 mm segments, and soaked in 25 mL of 95% ethanol for 3 days at 25°C. Supernatant absorbance was measured at 665 nm and 649 nm wavelengths using the UV spectrophotometer, and chlorophyll content was calculated using the following formula: C_a_ = 13.95 × A_665_ − 6.88 × A_649_; C_b_ = 24.96 × A_649_ – 7.32 × A_665_; C_t_ = (C_a_ + C_b_) = 18.08 × A_649_ + 6.63 × A_665_.

### Malondialdehyde

We used UV spectrophotometry to measure malondialdehyde (MDA), according to the method described by Cui and Wang (2006) with slight modifications. To do this, wheat leaves were homogenized in 10% (w/v) trichloroacetic acid (TCA) with a pestle and mortar before being centrifuged. The supernatant was then mixed with the same volume of thiobarbituric acid (TBA) (i.e., 0.6% TBA in 10% TCA), heated for 15 min in a boiling water bath, and cooled rapidly. The absorbance of the supernatant was measured at 450 nm, 532 nm and 600 nm. MDA concentration was calculated using the following formula: C = 6.45 × (A_532_ − A_600_) − 0.56 × A_450_.

### Electrolyte leakage rate

The relative electrolyte leakage rate was measured according to the method described by Guo et al. (2017) with slight modifications. To do this, the middle section of the second leaf from four uniform seedlings was cut into equal-sized pieces (5 cm long) and placed in 10 mL of distilled water. Next, the leaf pieces were boiling in a vacuum desiccator for 30 min, and then shaken for 4 h. The electric conductivity of the leaves was measured before and after boiling (S1 and S2), and compared to that of distilled water (S0). The electrolyte leakage rate was then calculated using the following formula: (S1 − S0)/(S2 − S0) × 100%.

### Detection and measurement of superoxide anions (${\text{O}}_{2}^{\cdot -}$)

We used nitro-blue tetrazolium **(**NBT) staining aids for visual detection of superoxide anions (${\text{O}}_{2}^{\cdot -}$) (Dutilleul et al., 2003). To achieve this, plant tissues were stained in a test tube with 10 mL NBT solution (i.e., 6 mM NBT supplied in a 50 mM HEPES buffer solution [pH = 7.5]) for 2 h in the dark, and chlorophyll was de-colorized from stained leaves by boiling them in 95% ethanol. De-stained leaves were then cleaned with chlorhydrate (i.e., 10 mL of water containing 25 g chlorhydrate), and photographed using a camera (Canon EOS 70D, Japan) microscope (OLYMPUS BX-53, Japan), before ${\text{O}}_{2}^{\cdot -}$ anions were extracted and measured following the method described by Wang S. et al. (2012).

### Detection and measurement of hydrogen peroxide

We used diaminobenzidine (DAB) staining method as described by Dutilleul et al. (2003) with slight modifications to visually detect hydrogen peroxide (H_2_O_2_). To achieve this, 3 cm leaf segments were placed into test tubes containing 10 mL of 3,3-DAB solution (i.e., 5 mM DAB supplied in a 10 mM 2-N-Morpholino ethanesulfonic acid buffer solution [MES, pH = 3.8]) for 15 h in the dark. Subsequent steps were as described for NBT staining, while H_2_O_2_ was extracted and measured following the method described by Brennan and Frenkel (1977).

### Antioxidant enzyme activity

We prepared enzyme extraction according to the method as described by Guo et al. (2017) with minor modification as follows: wheat leaves (0.5 g) were ground into powder with liquid nitrogen, and 2 mL of ice-cold 0.2 M potassium phosphate buffer (i.e., pH = 7.8 with 0.1 mM ethylenediaminetetraacetic acid, EDTA) was added and mixed to a homogenate. Mixtures were then centrifuged at 10,000 × g for 20 min at 4°C, and the crude supernatant extract was used to assay enzyme activities.

We measured catalase (CAT) activity according to the method of Aebi (1984) with slight modification. Enzyme extraction (100 μL) was added to a 3 mL reaction solution (1 L PBS [0.15 M, pH = 7.0] with 1.046 mL 30% H_2_O_2_), and absorbance at 240 nm was recorded every 30 s for 2 min. CAT activity was calculated using the following formula: (ΔA_240_ × Vt) / (W × V × 0.01 × t), where Vt is the total volume of supernatant extract, W is the fresh weight of the sample, V is the volume of extract for the reaction, and “t” is the reaction time.

Peroxidase (POD) activity was measured using the improved method of Hammerschmidt et al. (1982). Enzyme extraction (30 μL) was added into a 3 mL reaction solution (1 L PBS [0.2 M, pH = 6.0] with 0.36 mL guaiacol and 0.506 mL 30% H_2_O_2_), and absorbance at 470 nm was again recorded every 30 s for 2 min. POD activity was calculated using the following formula: (ΔA_470_ × Vt)/(W × V × 0.01 × t). Vt is the total volume of supernatant extract, W is the fresh weight of sample, V is the volume of extract for the reaction, and “t” is the reaction time.

### The detection of cell death

We used trypan blue staining to visually detect cell death according to Mason et al. (2016). To do this, leaf segments were stained in a test tube containing 0.04% lactophenol-trypan blue solution (Sigma-Aldrich, USA) dissolved in 10 mL of distilled water and diluted 1:1 with ethanol. After boiling for 1 min and incubated in staining solution for 5 to 10 min, the samples were removed from the staining solution and placed in a 10 mL de-staining buffer (i.e., 2.5 g/mL chloral hydrate) for 2 h on an orbital shaker. The leaf segments were further de-stained overnight using new buffer before being examined under the microscope. Numbers of stained cells and total cells in a representative view were counted, and the cell death rate was calculated by dividing the number of stained cells by the number of total cells.

### Analysis of ER stress–related gene expression

A quantitative real-time PCR (polymerase chain reaction) method was used to analyze the gene expression. After 0, 1, 3, 6, 12, 24, and 48 h of PEG stress treatment, the leaf samples were collected for RNA extraction. Total RNA was extracted using TranZol Up (Transgen, USA) and synthesized complementary DNA by means of the GoScript Reverse Transcription System (Promega, USA) following the manufacturer's instructions. We then conducted quantitative real-time PCR assays using a GoTaq qPCR Master Mix (Promega, USA) on a Thermal Cycler CFX96 Real-Time System (Bio-Rad, USA). The primers used for the ER stress response genes are listed in Table S1. A two-step fast PCR amplification procedure was used for qPCR, and related gene expression was calculated using the 2^−ΔΔCt^ method, as follows: (1) the Ct value of the gene was normalized against the internal reference gene (β-actin), and ΔCt was calculated; (2) the ΔCt of the gene under different treatments was normalized against the control to calculate ΔΔCt; (3) the 2^−ΔΔCt^ value was then calculated to demonstrate the fold changes of gene expression under different treatments (Livak and Schmittgen, 2001).

### Statistical analyses

Each data point in this study was represented as the mean ± standard deviation (SD) of three or four biological replicates. The statistical significance of differences in the measured parameters was tested using SPSS 16.0 software (SPSS Inc., Chicago, IL, USA); *P*-values were calculated using one-way analysis of variance (ANOVA), with *P* < 0.05 considered significant.

## Results

### TUDCA alleviates the impact of osmotic stress on wheat growth and development

**Figure 1:**
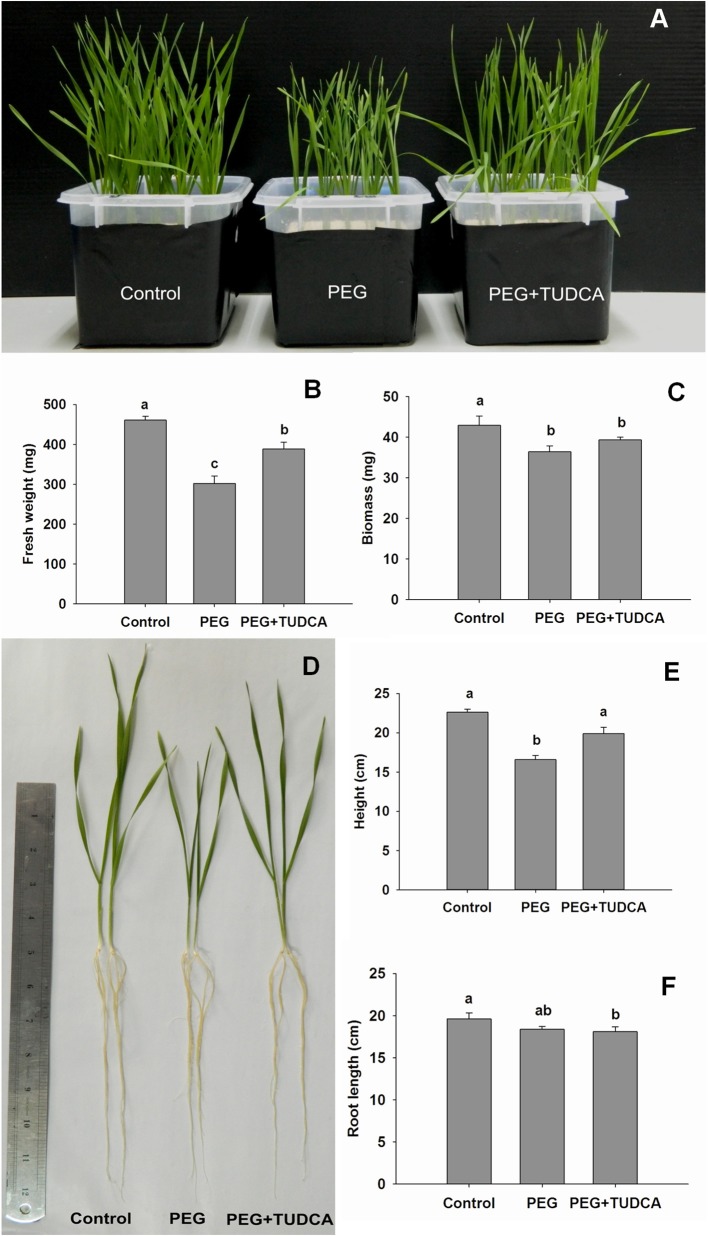
**Morphological changes in wheat seedlings under different treatments. (A)** Seedling features of control, PEG, and PEG + TUDCA (100 μg/mL) treatment groups after 4 days; **(B**,**C)** Seedling fresh weight and biomass accumulation of the three treatment groups after PEG treatment for 4 days; **(D)** Individual seedling features of different treatment groups after 4 days; **(E,F)** Average seedling height and root length of different treatment groups after 4 days. Data are shown as mean ± SD (*n* = 4) of three independent experiments. Different letters (a, b, or c) indicate significant difference between the groups (*P* < 0.05).

To assess whether TUDCA participated in the PEG-induced osmotic stress responses of wheat, we observed and analyzed seedling growth and development. The results showed that 20% PEG stress significantly influenced the growth and development of wheat seedlings, especially the above-ground section (Figures 1A,D), while seedling height and fresh and dry weight accumulation markedly decreased (Figures 1B–D). However, pre-treatment with TUDCA alleviated the impact of osmotic stress on the growth of wheat seedlings (Figure 1). Compared with 20% PEG stress in the absence of TUDCA, pre-treatment under osmotic stress increased the fresh weight as well as the height by 28.5 and 10.86%, respectively. Phenotype differences between the three treatment groups clearly showed that inhibition of seedling growth caused by osmotic stress was significantly alleviated by TUDCA (Figure 1).

### TUDCA relieves degradation of chlorophyll content, reduces membrane injury, and improves the antioxidant enzyme activity induced by osmotic stress in wheat

**Figure 2:**
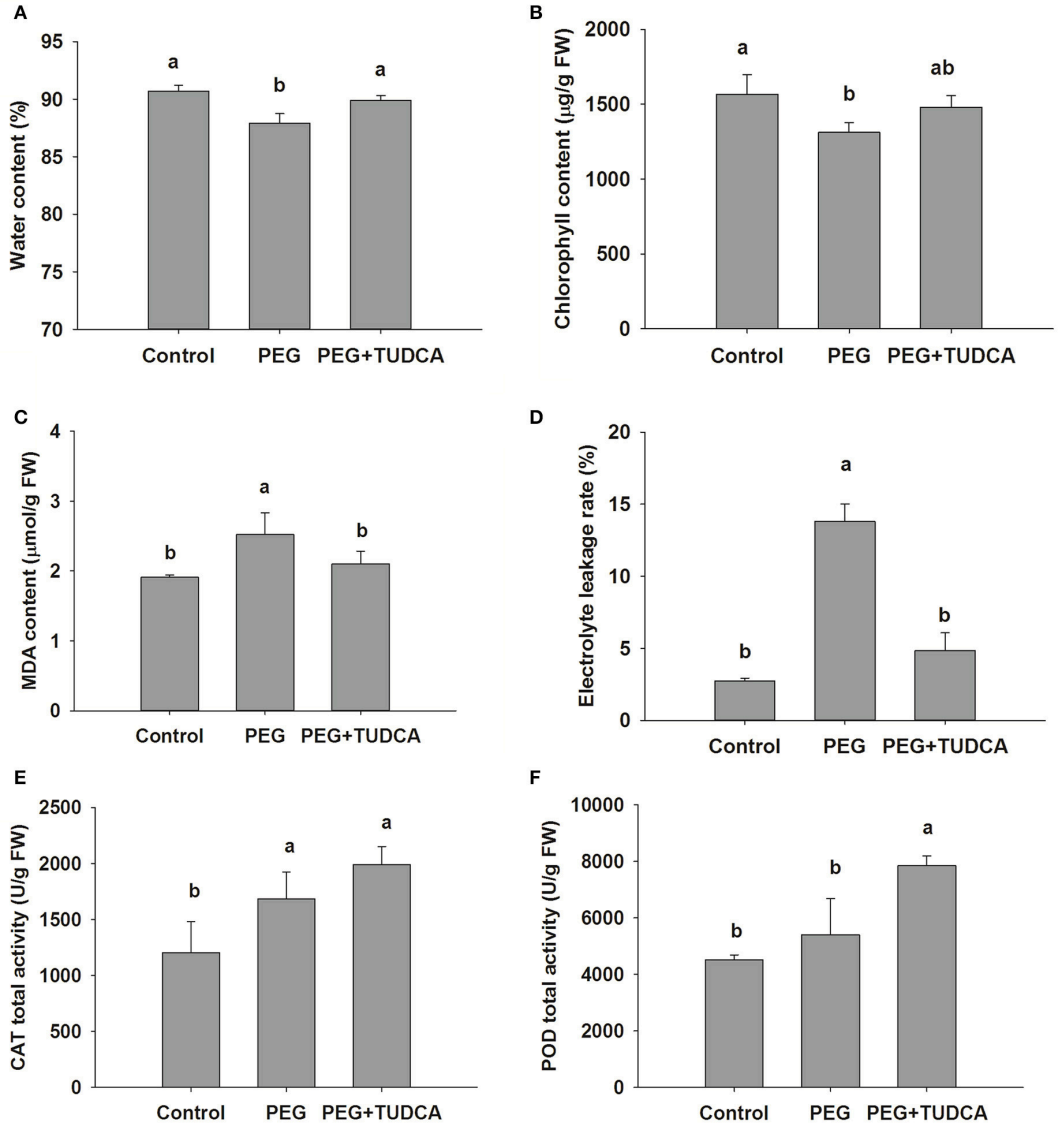
**Physiological changes in wheat leaves under different treatments. (A,B)** Leaf water content and chlorophyll content of control, PEG, and PEG + TUDCA (100 μg/mL) groups after 4 days of treatments; **(C,D)** MDA content and electrolyte leakage (%) in leaves after 8 days of treatments; **(E,F)** CAT and POD activities in leaves after 4 days of treatments. Data are shown as mean ± SD (*n* = 4). Different letters (a or b) indicate significant difference between the groups (*P* < 0.05).

We conducted a series of further experiments on wheat to understand the physiologically regulated mechanisms of TUDCA. The results of these physiological experiments showed that the water and chlorophyll content of the wheat seedlings were reduced by 3.04 and 16.23%, respectively, following PEG treatment for 4 days, while our two indicators of TUDCA pre-treatment showed no significant differences compared with the control group (Figures 2A,B). We further investigated the TUDCA effects on chlorophyll a and b content and found that both of them exhibited the same pattern as total chlorophyll (Figure S7). These results clearly showed that TUDCA alleviated the degradation of chlorophyll and water content induced by the PEG-simulated osmotic stress.

We measured variations in MDA content, electrolyte leakage, and membrane oxidation products and traits in wheat seedlings as these are all important indicators of membrane damage during osmotic stress. Our results showed that, as expected, when leaves were exposed to 20% PEG for 8 days, electrolyte leakage and MDA content increased by 32.0 and 185.2%, respectively, and that both trends were reversed following pre-treatment with TUDCA (Figures 2C,D). Thus, our results demonstrated that TUDCA effectively reduced the membrane injuries caused by osmotic stress.

It is well known that the antioxidant enzyme system plays a vital role in the removal of ROS in plants. Because both POD and CAT, two important antioxidant enzymes, influence the degree of oxidative damage caused to cells as well as a plant's degree of resistance, we measured their activities in this study. Our results showed that osmotic stress promotes the activities of both POD and CAT in wheat leaves, and that both were further enhanced by the PEG + TUDCA pre-treatment group (Figures 2E,F). Based on the time course changes in H_2_O_2_ content and CAT activity in leaves (Figure S6), we speculate that the remission of the membrane injury caused by TUDCA under the PEG stress might have been associated with further increases in antioxidant enzyme activity in the PEG + TUDCA pre-treatment groups.

### TUDCA alleviates oxidative damage to wheat induced by osmotic stress

We conducted a series of qualitative and quantitative analyses of ROS to further explore the reasons why TUDCA alleviates the damage to wheat cells caused by osmotic stress. We examined the conditions underlying the accumulation of ${\text{O}}_{2}^{\cdot -}$ and H_2_O_2_ in wheat tissues by using NBT and DAB staining, respectively. This technique takes advantage of the fact that ${\text{O}}_{2}^{\cdot -}$ reacts with NBT staining solution and forms dark blue spots, which can then be observed on plant tissue; the higher the number of blue spots, the higher the level of ${\text{O}}_{2}^{\cdot -}$ accumulation. Similarly, H_2_O_2_ reacts with DAB staining solution and forms orange marks that can also be observed on plant tissue; the deeper the color of the orange spots, the higher the level of H_2_O_2_ accumulation.

**Figure 3:**
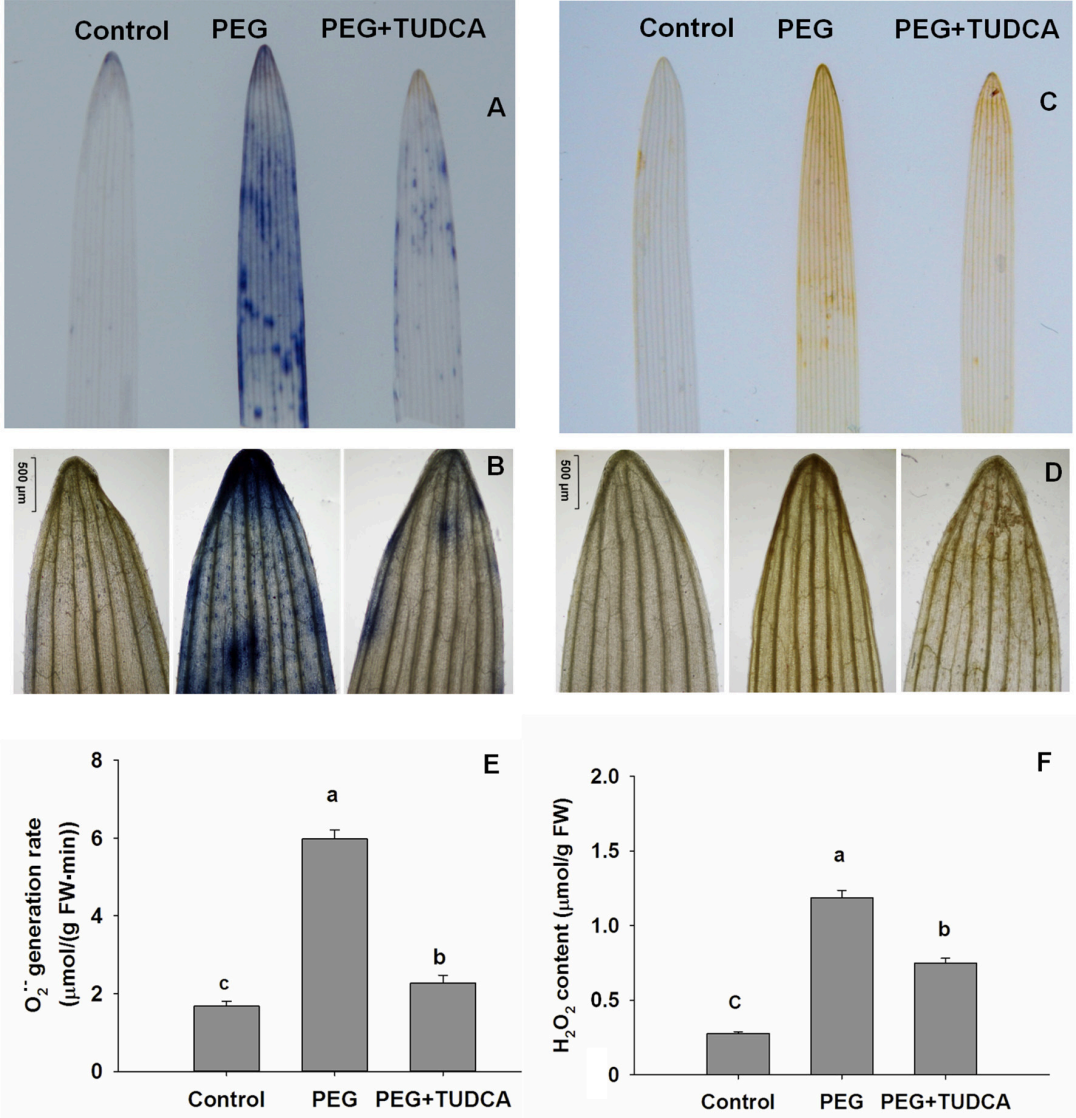
**ROS accumulation in wheat leaves under different treatments. (A,B)** ${\text{O}}_{2}^{\cdot -}$ accumulation in wheat leaves under control, PEG, and PEG + TUDCA groups after 5 days of treatment. Measurements were conducted via NBT staining, and photographed using a camera and ×40 magnification. A higher number of blue spots reflects a higher accumulation of ${\text{O}}_{2}^{\cdot -}$ **(C,D)** H_2_O_2_ accumulation in wheat leaves under control, PEG, and PEG + TUDCA groups after 5 days of treatment. Levels were detected by DAB staining and photographed using a camera and ×40 magnification. A deeper brown spot color shows a higher accumulation of H_2_O_2_; **(E,F)** Comparison of ${\text{O}}_{2}^{\cdot -}$ production rate and H_2_O_2_ content in leaves of different groups after 5 days of treatment. Data are shown as mean ± SD (*n* = 3). Different letters (a, b, or c) indicate significant difference between the groups (*P* < 0.05).

**Figure 4:**
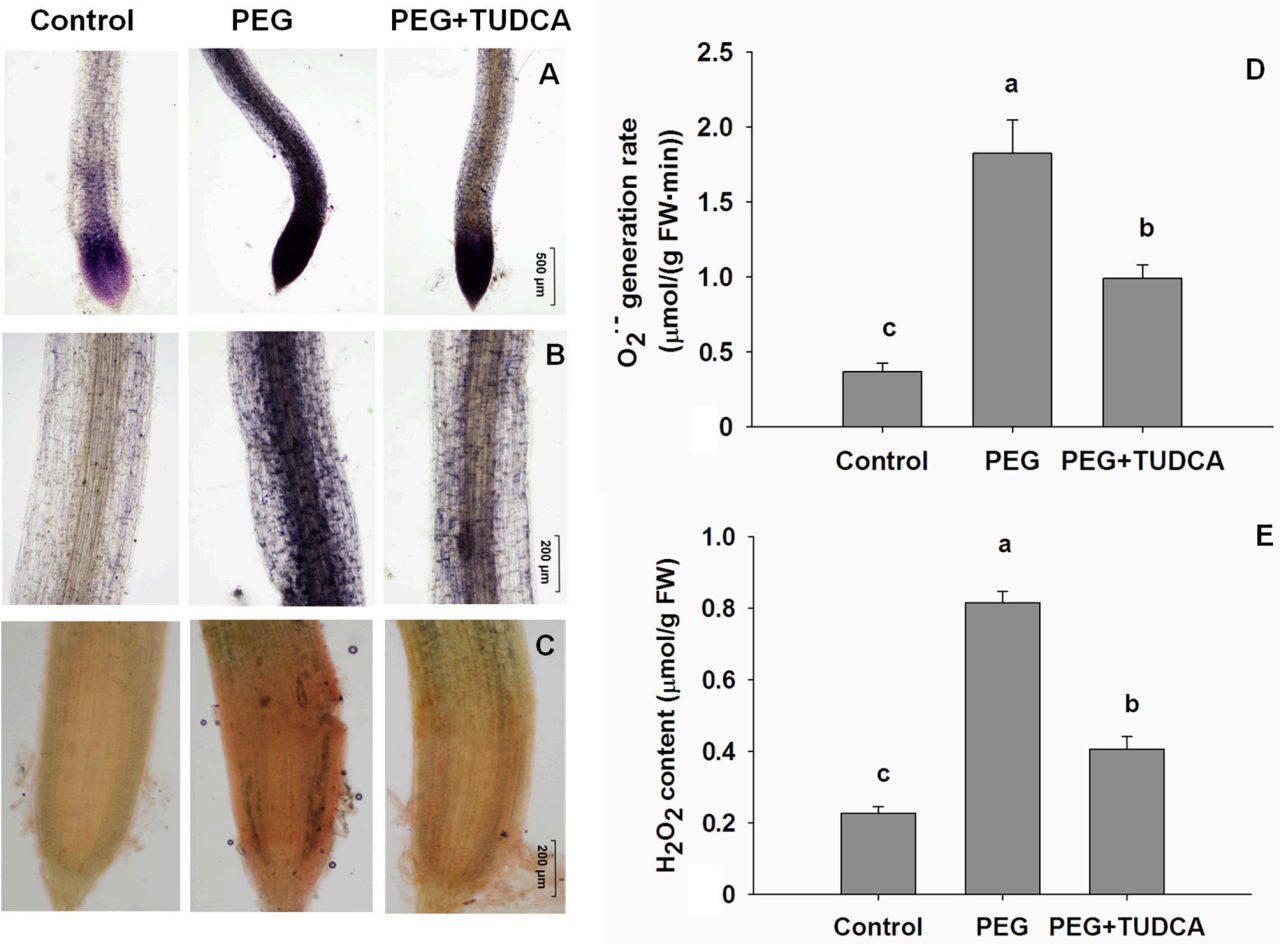
**ROS accumulation in wheat roots under different treatments. (A,B)** ${\text{O}}_{2}^{\cdot -}$ accumulation in wheat roots under control, PEG, and PEG + TUDCA groups after 5 days of treatment. Levels were indicated via NBT staining and photographed using a microscope (**A**, × 40 magnification; **B**, × 100 magnification). A deeper blue color indicates a higher accumulation of ${\text{O}}_{2}^{\cdot -}$; **(C)** H_2_O_2_ accumulation in wheat roots under control, PEG, and PEG + TUDCA groups after 5 days of treatment. Levels were indicated via DAB staining and photographed using a microscope (**C**, × 100 magnification). A deeper brown color indicates a higher accumulation of H_2_O_2_; **(D**,**E)** ${\text{O}}_{2}^{\cdot -}$ production rate and H_2_O_2_ content in wheat roots under different groups after 5 days of treatment. Data are shown as mean ± SD (*n* = 3). Different letters (a, b, or c) indicate significant difference between the groups (*P* < 0.05).

As expected, the osmotic stress simulated by the 20% PEG caused an increase in the accumulation of ROS in both wheat leaves and roots after 5 days of treatment. Color markers for ${\text{O}}_{2}^{\cdot -}$ and H_2_O_2_ were obvious on wheat leaves and roots following 20% PEG stress (Figures 3, 4), while the color of the stained wheat tissues in the PEG + TUDCA pre-treatment groups was significantly lighter (Figures 3A–D, 4A–C). The results clearly demonstrated that TUDCA significantly alleviated the accumulation of ROS induced by the osmotic stress.

Our quantitative analyses to determine variation in ${\text{O}}_{2}^{\cdot -}$ production rate and H_2_O_2_ content revealed the same trends as our qualitative approach. Compared with the control, the ${\text{O}}_{2}^{\cdot -}$ production rate of wheat leaves and roots increased dramatically—by 3.57-times and 4.94-times, respectively—following treatment with 20% PEG, while the increase in the PEG + TUDCA pre-treatment groups was less marked—with leaves and roots increasing by just 1.36-times and 2.69-times, respectively (Figures 3E, 4D). The H_2_O_2_ content of the wheat leaves and roots increased by 4.31-times and 3.58-times, respectively, under osmotic stress, while pre-treatment with TUDCA almost entirely counteracted the increase in H_2_O_2_ in both parts of the plant under PEG stress (Figures 3F, 4E).

### TUDCA restrains wheat cell death induced by osmotic stress

**Figure 5:**
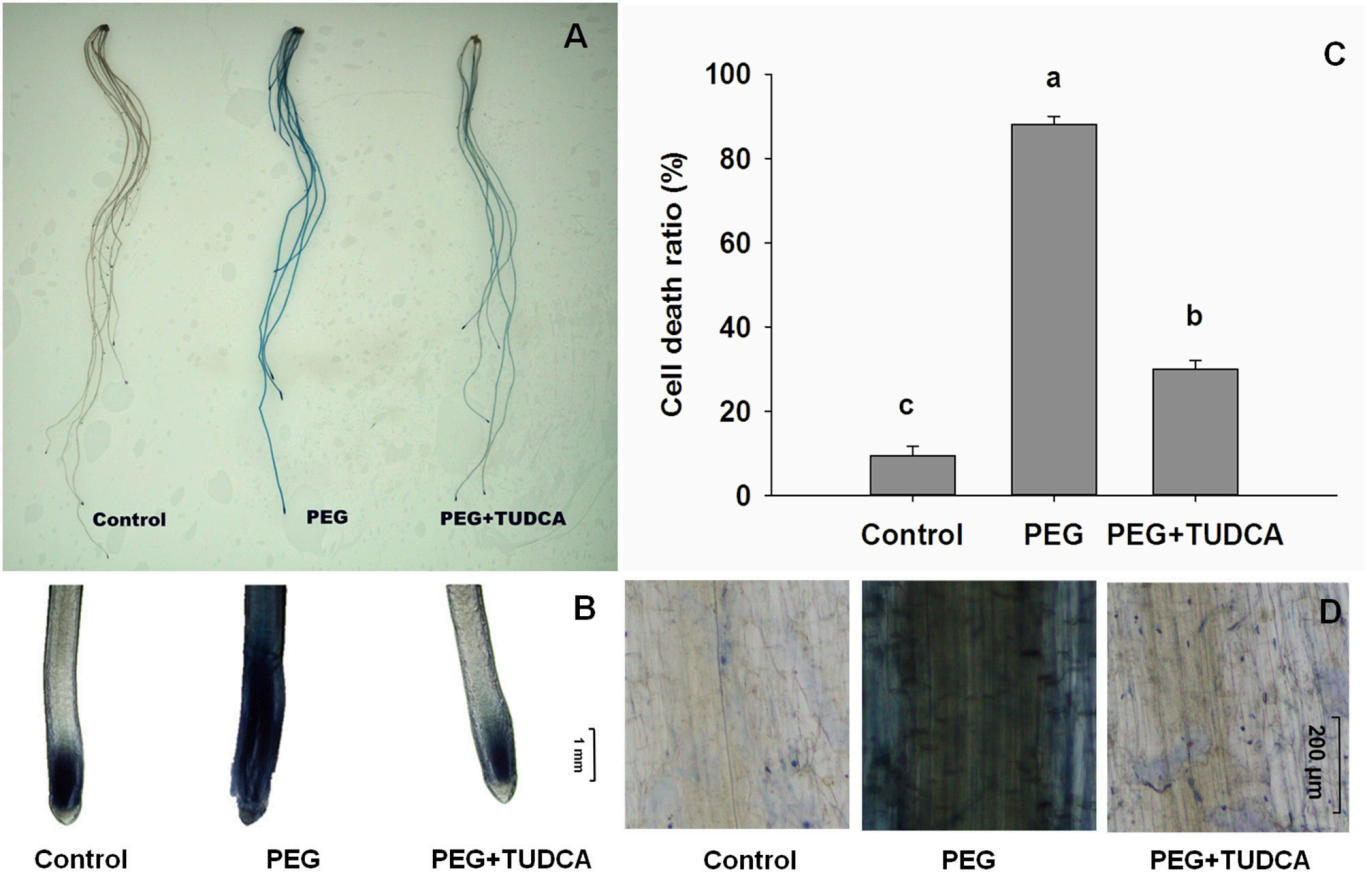
**Status of cell death in wheat roots under different treatments. (A,B,D)** Dead cells in wheat roots under control, PEG, and PEG + TUDCA groups after 5 days of treatment. Root tissues were stained with trypan blue solution and photographed with a camera **(A)**, and microscope (**B**, ×40 magnification; **D**, ×100 magnification); **(C)** Comparison of cell death ratio in roots under three different treatments. Data are shown as mean ± SD (*n* = 3). Different letters (a, b, or c) indicate significant difference between the groups (*P* < 0.05).

As in most cases, severe and prolonged ER stress always results in cell death, and the severity of this effect is directly related to the conditions. Thus, to assess the effects of TUDCA on ER stress in wheat under osmotic stress conditions, the extent of cell death extent in the different treatments groups was evaluated using trypan blue staining. This chemical enters dead cells, staining them blue, but cannot enter living cells because of the presence of the intact cell membrane. The results of this treatment showed that the wheat roots subjected to PEG treatment were obviously stained blue, while the control specimens barely changed color, and that the roots subjected to the PEG + TUDCA pre-treatment were stained blue to a small degree (Figure 5A). Our microscopic examination of root cells illustrated the same trend (Figures 5B,D); while prolonged exposure to osmotic stress caused serious cell death in the wheat roots, this was alleviated by treatment with TUDCA.

To further evaluate cell death and ER stress–related conditions, we calculated the death ratios resulting from different treatments. The results of this analysis showed that the cell death ratios of the wheat roots in the control treatments were less than 9.3%, while those that resulted from PEG treatment were always 85% or higher. The wheat root tip cell death ratio following the TUDCA pre-treatment was significantly less than that following the PEG treatment under osmotic stress, with a cell death ratio of not more than 30% (Figure 5C), suggesting that TUDCA pre-treatment can greatly reduce the extent of cell death induced by osmotic stress.

### TUDCA regulates the expression of ER stress–related gene expression under osmotic stress

To gain further insights into the mechanisms underlying TUDCA-mediated relief of ER stress caused by osmotic stress, we determined the expression levels of ER stress–related genes such as *bZIP60, BiP, PDIL, and GRP94*. Our results showed that the genes regulated by TUDCA over certain periods under osmotic stress can be divided into early- (e.g., *bZIP60B* and *bZIP60D*), interim- (e.g., *BiP1* and *BiP2*), and late-response (e.g., *GRP94* and *PDIL8-1*) genes.

**Figure 6:**
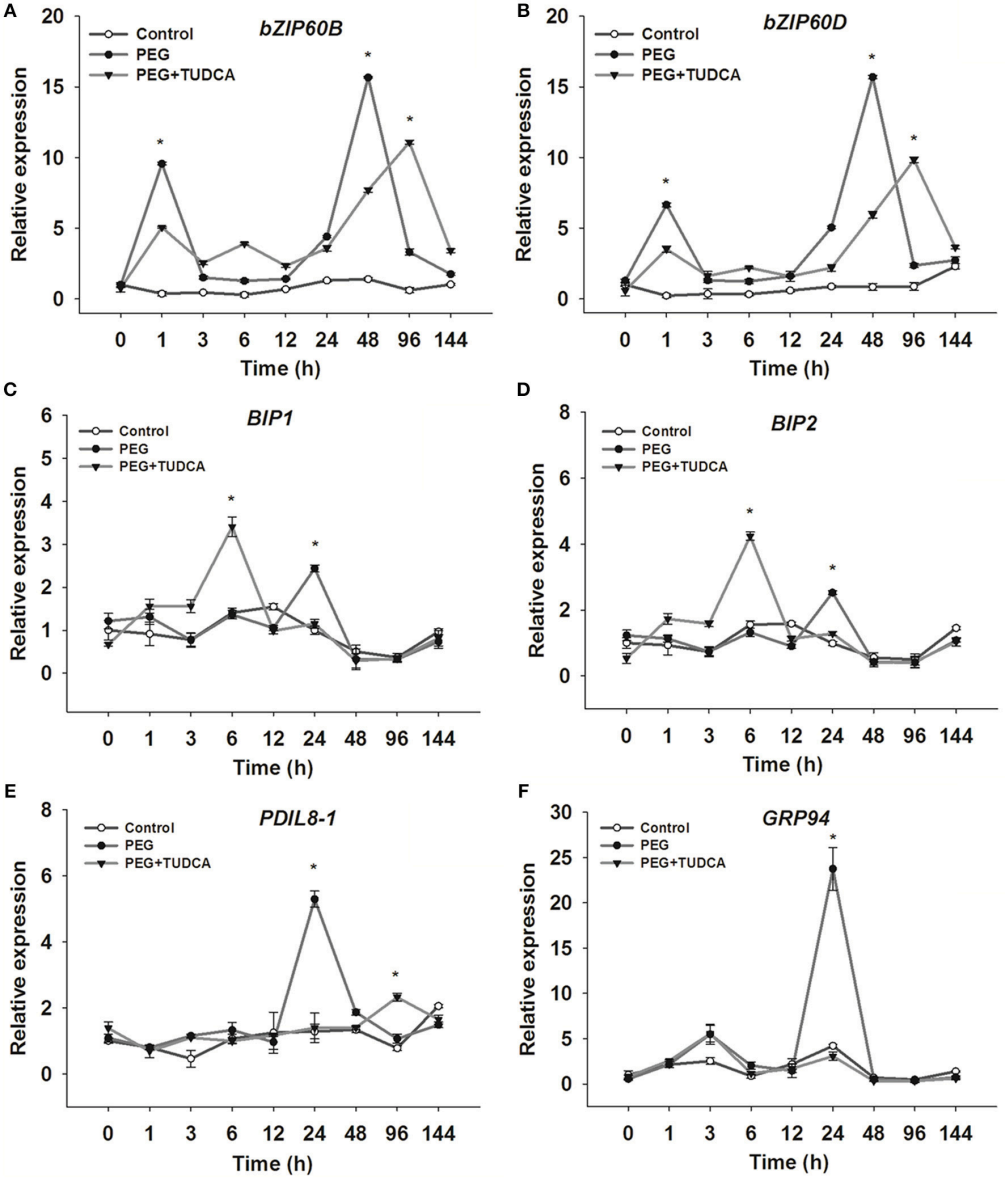
**Relative expression of ER stress–related genes in wheat leaves under different treatments. (A,B)** Relative expression of *TabZIP60B* and *TabZIP60D*, two typical early response wheat genes; **(C,D)** Relative expression of *BiP1* and *BiP2*, two typical interim response wheat genes; **(E,F)** Relative expression of *PDIL8-1* and *GRP94*, two typical late response wheat genes. Data are shown as mean ± SD (*n* = 3) of three independent experiments. Significant difference between the groups (*P* < 0.05) is indicated by an ^*^.

It is well known that bZIP60s are important plant transcription factors that participate in the expression and regulation of ER stress-related and other genes in Arabidopsis (Sabrina et al., 2012; Henriquez-Valencia et al., 2015). The results of this study showed that both TabZIP60B and TabZIP60D respond to osmotic stress very quickly. After 1 h of PEG stress, the relative expression of *bZIP60B and bZIP60D* was obviously up-regulated in the PEG and PEG + TUDCA treatment groups compared with the control, and after 48 h of PEG stress, their expression was up-regulated again, while the expression of these two genes in the PEG + TUDCA treatment group was significantly lower or later than that in the PEG treatment group (Figures 6A,B). Clearly, TUDCA pre-treatment suppresses or delays the increase in *TabZIP60B* and *TabZIP60D* gene expression under osmotic stress.

BiP is an important molecular chaperone that confers plant stress resistance (Valente et al., 2009; Xu et al., 2013; Carvalho et al., 2014). In our study, the typical interim response genes BiP1 and BiP2 exhibited similar trends under both PEG stress and TUDCA pre-treatment. Compared with the control group, the relative expression levels of these two genes were obviously up-regulated after PEG stress for 24 h, while they were dramatically up-regulated 6 h after PEG treatment in the TUDCA pre-treatment group (Figures 6C,D). In other words, processing with TUDCA enables a faster stress response in wheat, significantly raising the expression levels of molecular chaperone BiP genes.

TaBI61 and TaBI85 are homologous to AtBI-1, which modulates ER stress–mediated programmed cell death in Arabidopsis (Watanabe and Lam, 2008). The results showed that TaBI61 and TaBI85 responded to PEG stress as well as TUDCA pre-treatment; the expression levels of these genes were obviously up-regulated when subjected to osmotic stress for 6 h, while up-regulation was suppressed by TUDCA (Figure S8).

The gene GRP94 is a member of the HSP90 family that is also activated by stress (Mishra et al., 2016), while PDIL8-1 is a member of the PDI family and catalyzes the formation of disulfide bonds in newly synthesized proteins within the ER lumen (Dong et al., 2016). Our results showed that the expression levels of *GRP 94* and *PDIL8-1* after 24 h of osmotic stress were significantly higher than those of the control and PEG + TUDCA treatment group, but there was no significant difference between the PEG + TUDCA treatment group and the control (Figures 6E,F). In other words, processing with TUDCA represses the up-regulation of GRP94 and PDIL8-1 expression when subjected to osmotic stress for 24 h.

## Discussion

**Figure 7:**
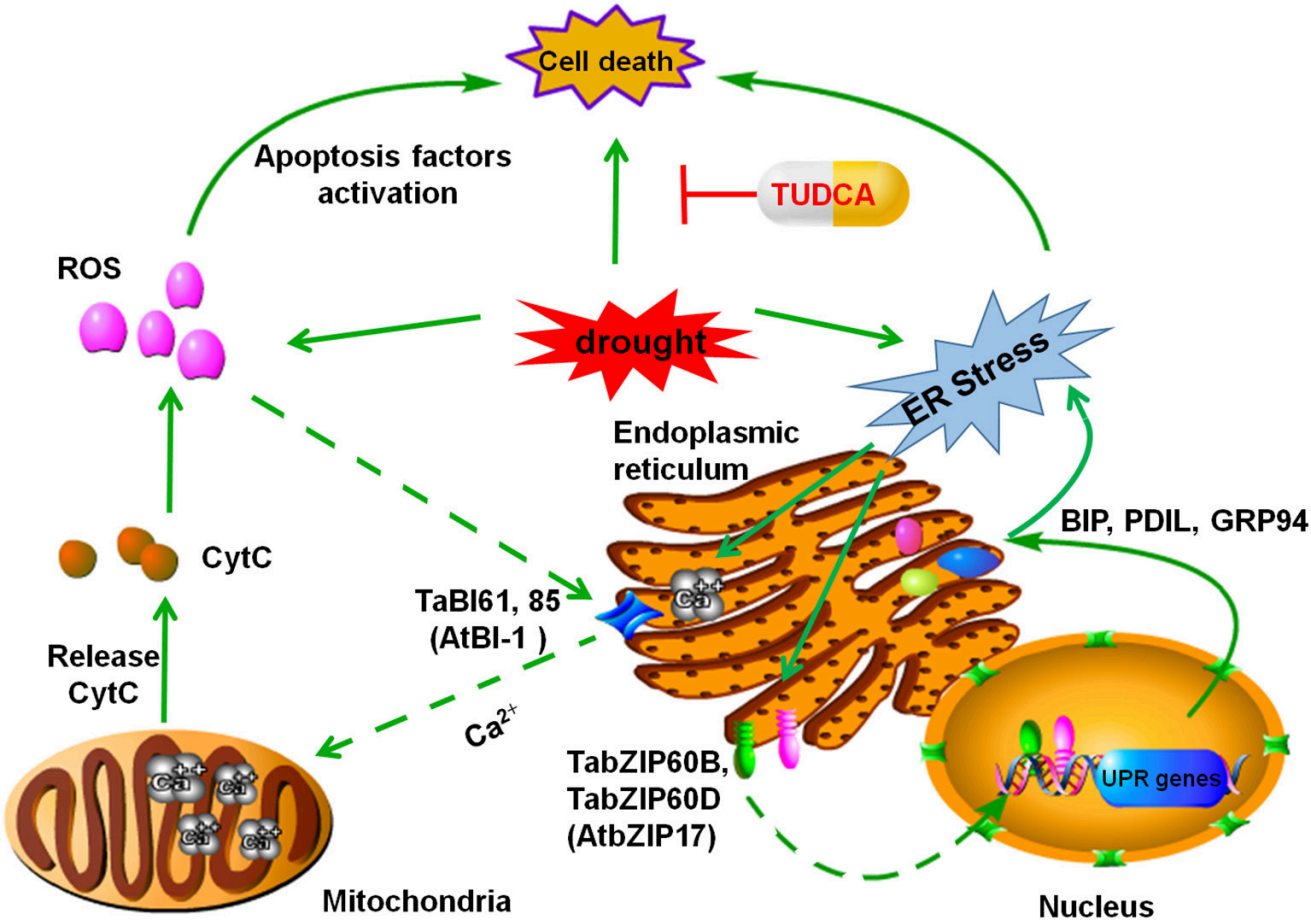
**Proposed ER stress–related pathway that leads to PCD in plants**. Under osmotic stress, orderly protein folding in the ER lumen is disturbed and results in ER stress. In one scenario, this stress activates the C-terminal Ca^2+^ channel in TaBI61 and TaBI85 (homologous proteins to AtBI-1) and leads to calcium efflux from the ER to the mitochondria, which in turn leads to Cyt C increase, ROS accumulation, and cell death. This process is referred to here as the ER stress–ROS–PCD pathway. In an alternative scenario, ER stress is sensed by the receptors TabZIPB and TabZIPD (homologous proteins to AtbZIP17), which transport ER stress signals to the nucleus and regulate UPR-related genes including TaBiP1, TaBiP2, TaPDIL8-1, and TaGRP94. This process is referred to here as the ER stress–UPR–PCD pathway. Treatment with TUDCA could prevent, or alleviate, the occurrence of wheat cell death induced by osmotic stress via either of these two ER stress–related pathways.

Based on our findings and previous studies in related fields, we proposed a model to explain how TUDCA alleviates wheat cell death caused by osmotic stress. In this model, we speculated that two ER stress-related pathways are involved in the regulation. One pathway is the ROS–cell death pathway, which is based on the effects of TUDCA on ROS accumulation in wheat tissues, while the other is the UPR–cell death pathway, which is based on the regulatory effect of TUDCA on ER stress and UPR-related gene expression under osmotic stress (Figure 7). This model provides a reasonable explanation of the regulative mechanisms of TUDCA underlying cell death that occur as a result of osmotic stress.

Previous studies have shown that, under ER stress, the C-terminal Ca^2+^ channel of the Bax inhibitor-1 (BI-1) gene is activated, and Ca^2+^ ions efflux from ER to the mitochondria, while calcium overload in the mitochondria enhances the release of cytochrome C (Cyt C) and decreases Cyt C oxidase (CCO) activity (Xu and Reed, 1998). This cascade leads directly to the accumulation of ROS (Williams et al., 2014; Petrov et al., 2015; Kobylinska and Posmyk, 2016). ROS accumulation in cells may activate apoptosis-inducing factors and result in programmed cell death (Hwang et al., 2014; Petrov et al., 2015). We refer to this as the ER stress–ROS–cell death pathway. BI-1 is conserved in plants and animals (Ishikawa et al., 2011). In our study, the relative expressions of TaBI61 and TaBI85, homologous to AtBI-1, were up-regulated under PEG stress, and the TUDCA treatment decreased gene up-regulation. In addition, TUDCA also relieved ROS damage and alleviated the cell death induced by osmotic stress. Based on these data, we speculate that TUDCA reduces ROS accumulation and alleviates cell death induced by osmotic stress possibly by regulating the expression of TaBI61 and TaBI85.

In addition, previous research has shown that ER stress signals can activate a UPR which then regulates expression of many stress-related genes, enhances protein-folding ability, reduces the accumulation of malformed proteins, and alleviates the degree of ER stress (Turano et al., 2002; Jia et al., 2008; Liu and Howell, 2010a; Han et al., 2011; Wang S. J. et al., 2012; Zhu et al., 2014). The results presented here clearly demonstrate that cell death induced by PEG stress is alleviated by TUDCA. Moreover, studies have revealed that a series of UPR-related genes that also respond to osmotic stress are regulated by TUDCA, including the early responders *TabZIP*60*B* and *TabZIP*60*D*, genes homologous to *AtbZIP17*. These genes are rapidly activated, within 60 min of PEG stress treatment, while molecular chaperones, including *BiP1, BiP2, PDIL8-1*, and *GRP94*, are also regulated under PEG stress by TUDCA. These genes, however, are slower to respond than *TabZIP60*, taking up to several hours or longer. Thus, building on previous research and incorporating our results, we hypothesize that the transcription factors *TabZIP60B* and *TabZIP60D* are able to sense ER stress and transport signals to the nucleus to regulate the expression of UPR-related genes. We propose that this mechanism influences the levels of cell death induced by osmotic stress in wheat, while the pathway regulated by TUDCA comprises ER stress–UPR–cell death.

Previous research has shown that over-expression of *B****i****P* confers drought stress resistance in soybean (*Glycine max*) and tobacco (*Nicotiana tabacum*) plants (Valente et al., 2009). We, too, found that expression of *BiP* genes increases in response to osmotic stress, while TUDCA pre-treatment led *BiP1* and *BiP2* to respond more quickly to osmotic stress, perhaps contributing to the alleviation of ER stress and cell death at early stages. We also hypothesize that this might represent the early warning function of TUDCA.

Additional research has reported that the loss of a protein disulfide isomerase-like protein (PDIL) protein leads to ER stress in rice (Han et al., 2011). Indeed, *GRP94/HSP90* is involved in a range of diverse physiological processes including signal transmission and responses to heat and osmotic stress (Wang S. J. et al., 2012). Research has demonstrated that *HSP90* was quickly up-regulated as a result of heat and salt shock in *Dunaliella salina* (Wang S. J. et al., 2012), while expression of *GRP94/HSP90* remained stable under drought stress in pigeonpea (Cajanus cajan (L.) Millsp.) (Sinha et al., 2015). In our study, although expression of *PDIL8-1* and *GRP94* were significantly up-regulated by PEG stress by 24 h after stress, pre-treatment with TUDCA rendered expression stable within 24 h of the onset of PEG stress. However, it might also be the case that pre-treatment with TUDCA alleviates the ER stress induced by PEG stress within 24 h, ameliorating the need to up-regulate expression of the two typical late response genes, *PDIL8-1* and *GRP94*, to cope with osmotic stress.

Although, we have made some progress in elucidating the processes by which TUDCA alleviates cell death, a number of issues remain unclear. It has been reported that TabZIP60 is involved in the responses of Arabidopsis to many environmental stresses, e.g., drought, salt, cold and abscisic acid (Zhang et al., 2015). However, the regulatory network and mechanism are unknown in terms of how it modulates the signal pathway. For example, how are TabZIP60B and TabZIP60D transported from the ER membrane to the nucleus to regulate UPR-related genes in wheat? Are TaBiP1 and TaBiP2 the downstream genes that correspond to these transcription factors? If this is the case, then why are the trends we see in their changes in responses to PEG stress and pre-treatment similar to those seen in *TabZIP*60*B* and *TabZIP*60*D*? What are the functions of ER stress-related genes in terms of adversity resistance? These questions remain unclear, along with others, and will require further verification via sub-cellular localization and genetic-modification experiments.

The key ER stress alleviator TUDCA has been widely researched in mammals, and its effects on resistance to environmental stress in plants is certain to become an important area for future study. Our development of a model for TUDCA-regulated cell death in wheat under osmotic stress provides vital insights as well as a theoretical foundation for further research.

To date, the role of ER-stress–related genes in the process of plant response to environmental stresses needs further clarification and investigation, though several of these genes have been well studied in recent years. By genetic engineering and other modern biological techniques, combined with pharmacological approaches, related experiments should be conducted in subsequent research to fully understand the functions of these genes.

To conclude, we have shown that TUDCA is able to relieve the inhibition of wheat seeding growth as well as the degradation of chlorophyll content induced by osmotic stress. Indeed, TUDCA also reduces injury to membranes by increasing antioxidant enzyme activity and decreasing the accumulation of ROS under osmotic stress. It is also possible that this compound alleviates cell death in wheat induced by osmotic stress via the ER stress–cell death signaling pathway, promotes expression of the binding protein BIP, and represses the up-regulated expression of PDIL8-1 and the HSP 90 family member GRP94. To summarize, TUDCA effectively alleviates osmotic stress by regulating the expression of a series of ER stress–related genes in order to relieve ER stress–mediated ROS accumulation and cell death.

## Author contributions

LZ and ZX designed and conduct the trial, and drafted and revised the manuscript, they two have the equal contribution on the article. XY, MZ QC, ZL, and YN took part in the experiment. CM, WL, ZW, YR, and TL gave many advices during the research. In addition, TL provide financial support for the study.

### Conflict of interest statement

The authors declare that the research was conducted in the absence of any commercial or financial relationships that could be construed as a potential conflict of interest.

## Supplementary material

Figure S1 **Morphological changes in wheat seedlings under TUDCA treatment for 4 days. (A,C)** Seedling features of control and TUDCA treatment groups after 4 days of treatment; **(B–E)** Seedling biomass, heights, and root lengths of two treatment groups after 4 days. Data are shown as mean ± SD (*n* = 4) of three independent experiments. Different letters (a, b, or c) indicate significant difference between the groups (*P* < 0.05).

Figure S2 **Physiological changes in wheat leaves under TUDCA treatment for 4 days. (A,B,E)** Chlorophyll content, ${\text{O}}_{2}^{\cdot -}$ production rate and MDA content of control, TUDCA groups after 4 days of TUDCA treatment; **(C,D)** CAT and POD activities in leaves after TUDCA pre-treatment for 4 days. Data are shown as mean ± SD (*n* = 4). Different letters (a or b) indicate significant difference between the groups (*P* < 0.05).

Figure S3 **Time course of gene expression in wheat leaves under TUDCA treatment. (A,B)** Time course of bZIP60B and bZIP60D expression in wheat leaves under TUDCA treatment. Data are shown as mean ± SD (*n* = 3) of three independent experiments. Significant difference between the groups (*P* < 0.05) is indicated by an ^*^.

Figure S4 **Morphological changes in wheat seedlings under different treatments. (A,B,E,F)** Seedling features, height, and weight of control, PEG, and PEG + TUDCA (100 μg·mL^−1^) treatment groups after 2 days of PEG stress; **(C,D,G,H)** Seedling features, height, and weight of control, PEG, and PEG + TUDCA (100 μg·mL^−1^) treatment groups after 4 days of PEG stress. The red line in **(A,C)** is used to display difference in plant height. Data are shown as mean ± SD (*n* = 4). Different letters (a, b, or c) indicate significant difference between the groups (*P* < 0.05).

Figure S5 **Time course of MDA content in wheat leaves under different treatments**. MDA content in wheat leaves of control, PEG, and PEG + TUDCA (100 μg·mL^−1^) treatment groups after 0, 2, 4, and 8 days of PEG stress. Data are shown as mean ± SD (*n* = 4). Different letters (a or b) indicate significant difference between the groups (*P* < 0.05).

Figure S6 **Time course response of the CAT activity and H_2_O_2_ content in wheat leaves subjected to different treatments. (A)** CAT activity in wheat leaves of control, PEG, and PEG ± TUDCA treatment groups under PEG stress for 0, 2, 4, 6, and 8 days; **(B)** H_2_O_2_ content in wheat leaves of control, PEG, and PEG + TUDCA treatment groups under PEG stress for 0, 2, 4, 6, and 8 days. Data are shown as mean ± SD (*n* = 4) of three independent experiments. Significant difference between the groups (*P* < 0.05) is indicated by an ^*^.

Figure S7 **Chlorophyll content changes in wheat seedlings under different treatments**. Chlorophyll a, chlorophyll b and chlorophyll total content in wheat leaves of control, PEG, and PEG + TUDCA treatment groups under PEG stress for 4 days. Chl-a, chlorophyll a; Chl-b, chlorophyll b; Chl-t, total chlorophyll. Data are shown as mean ± SD (*n* = 4). Different letters (a, b or c) indicate significant difference between the groups (*P* < 0.05).

Figure S8 **Time course of BI61 and BI85 expression in wheat leaves. (A)** The relative expression of BI61 of control, PEG, and PEG + TUDCA treatment groups after 3, 6, and 12 h of PEG stress; **(B)** The relative expression of BI61 of control, PEG, and PEG + TUDCA treatment groups after 3, 6, and 12 h of PEG stress. Data are shown as mean ± SD (*n* = 3) of three independent experiments. Different letters (a or b) indicate significant difference between the groups (*P* < 0.05).
