# Supplementary figures and images for: Osmotic Stress Induced Cell Death in Wheat Is Alleviated by Tauroursodeoxycholic Acid and Involves Endoplasmic Reticulum Stress–Related Gene Expression
Source: Front Plant Sci. 2017 May 3;8:667. doi: 10.3389/fpls.2017.00667 (PMC5413500; doi:10.3389/fpls.2017.00667)

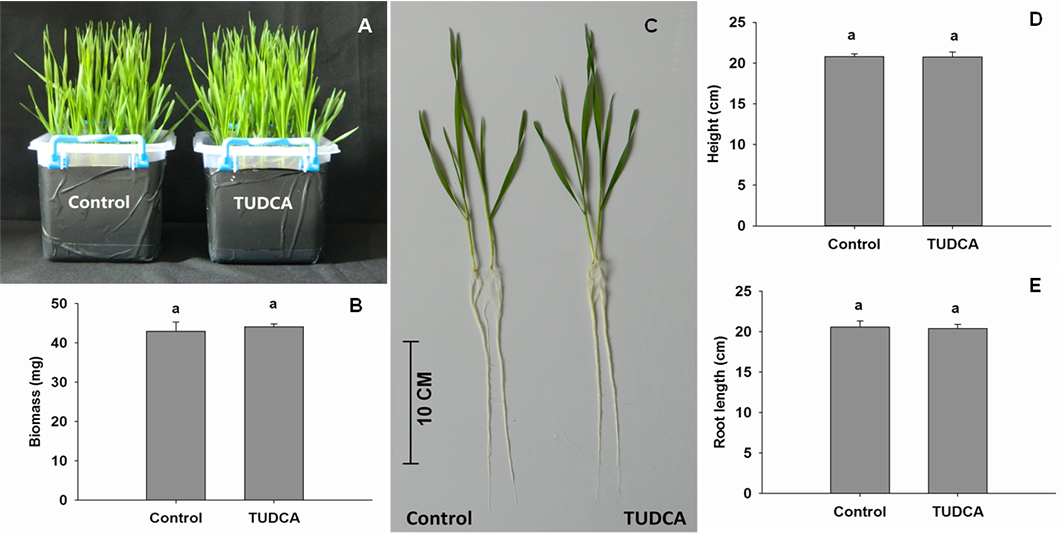

Supplement: Figure S1 — Morphological changes in wheat seedlings under TUDCA treatment for 4 days. (A,C) Seedling features of control and TUDCA treatment groups after 4 days of treatment; (B–E) Seedling biomass, heights, and root lengths of two treatment groups after 4 days. Data are shown as mean ± SD (n = 4) of three independent experiments. Different letters (a, b, or c) indicate significant difference between the groups (P < 0.05). [file Image1.TIF]

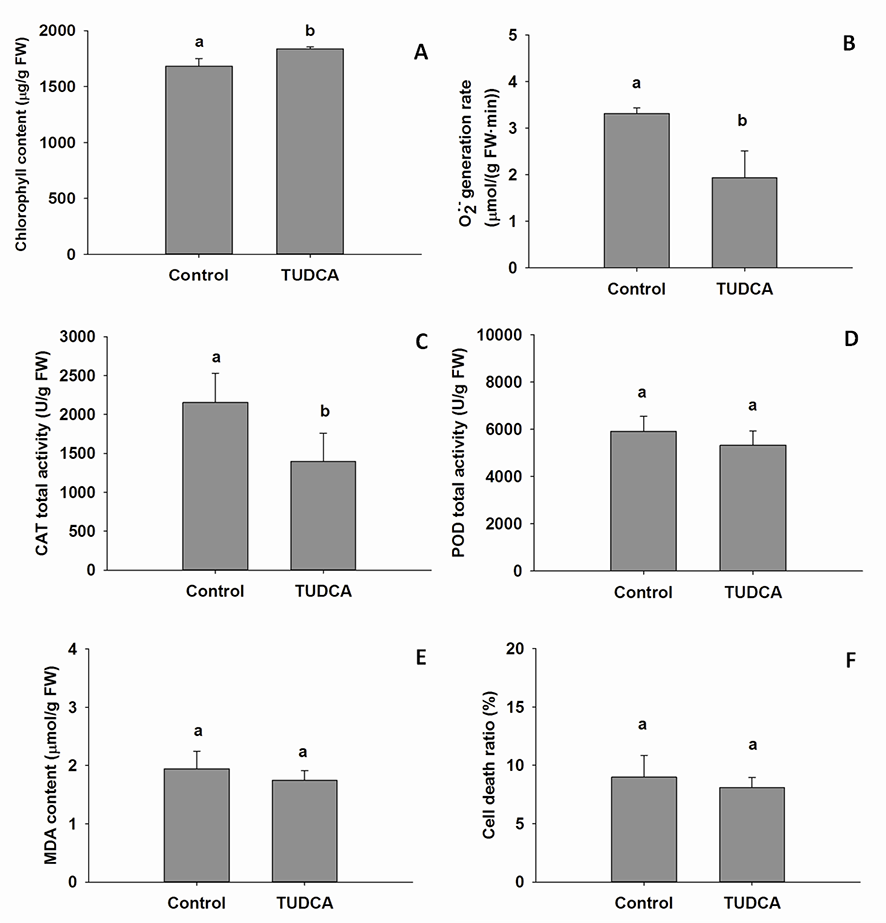

Supplement: Figure S2 — Physiological changes in wheat leaves under TUDCA treatment for 4 days. (A,B,E) Chlorophyll content, O2·- production rate and MDA content of control, TUDCA groups after 4 days of TUDCA treatment; (C,D) CAT and POD activities in leaves after TUDCA pre-treatment for 4 days. Data are shown as mean ± SD (n = 4). Different letters (a or b) indicate significant difference between the groups (P < 0.05). [file Image2.TIF]

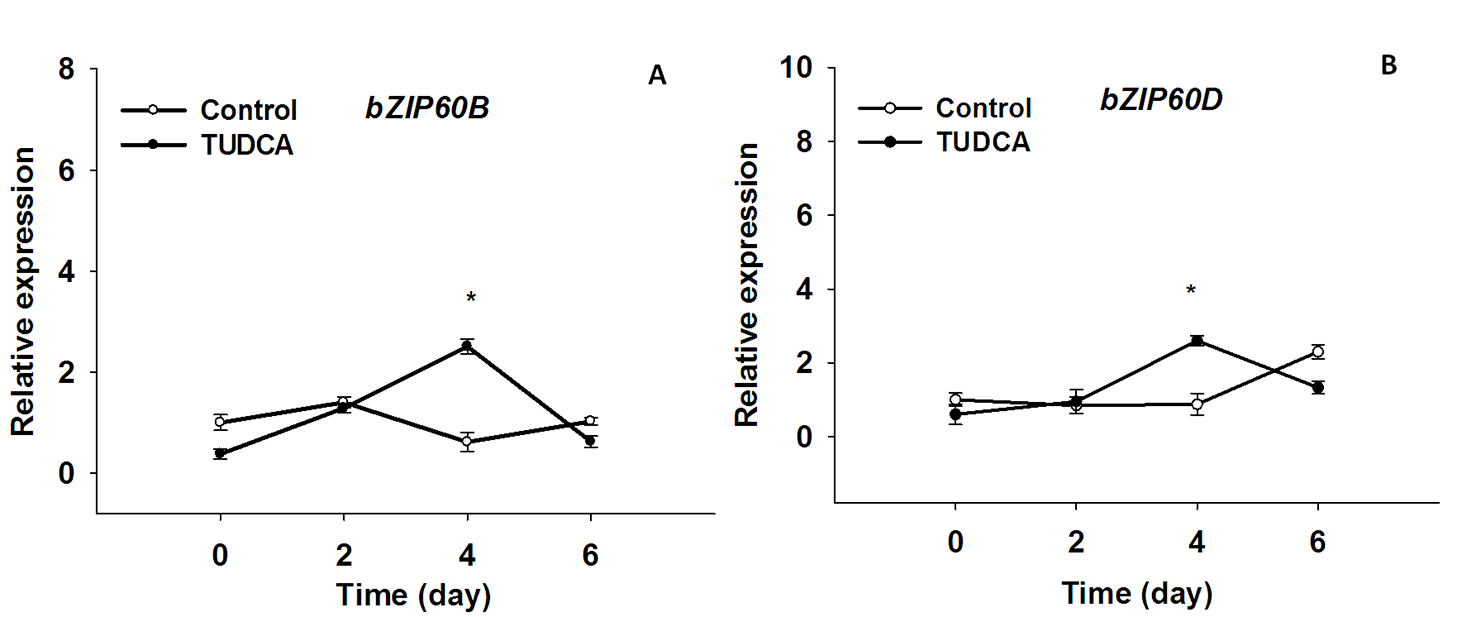

Supplement: Figure S3 — Time course of gene expression in wheat leaves under TUDCA treatment. (A,B) Time course of bZIP60B and bZIP60D expression in wheat leaves under TUDCA treatment. Data are shown as mean ± SD (n = 3) of three independent experiments. Significant difference between the groups (P < 0.05) is indicated by an *. [file Image3.TIF]

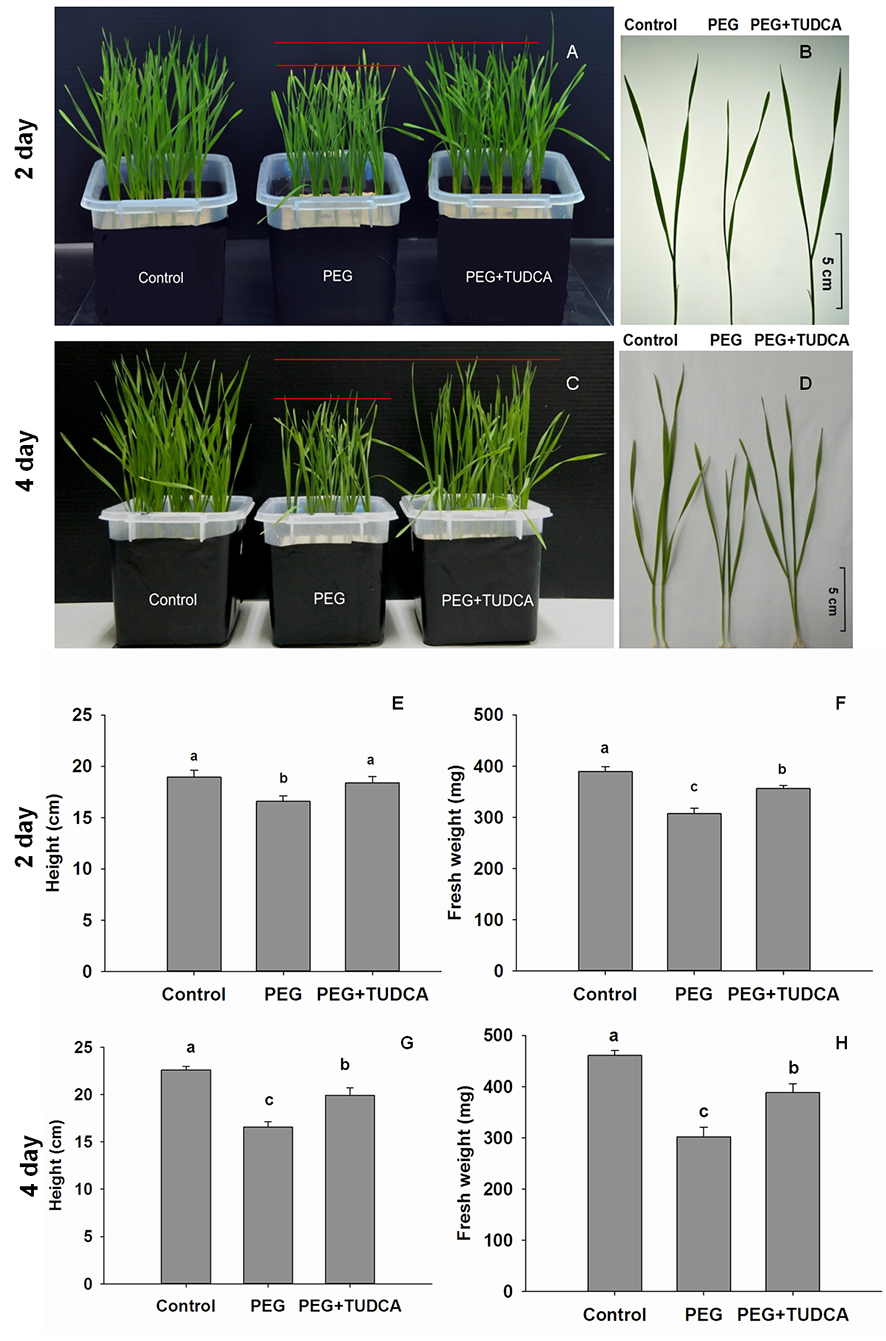

Supplement: Figure S4 — Morphological changes in wheat seedlings under different treatments. (A,B,E,F) Seedling features, height, and weight of control, PEG, and PEG + TUDCA (100 μg·mL−1) treatment groups after 2 days of PEG stress; (C,D,G,H) Seedling features, height, and weight of control, PEG, and PEG + TUDCA (100 μg·mL−1) treatment groups after 4 days of PEG stress. The red line in (A,C) is used to display difference in plant height. Data are shown as mean ± SD (n = 4). Different letters (a, b, or c) indicate significant difference between the groups (P < 0.05). [file Image4.TIF]

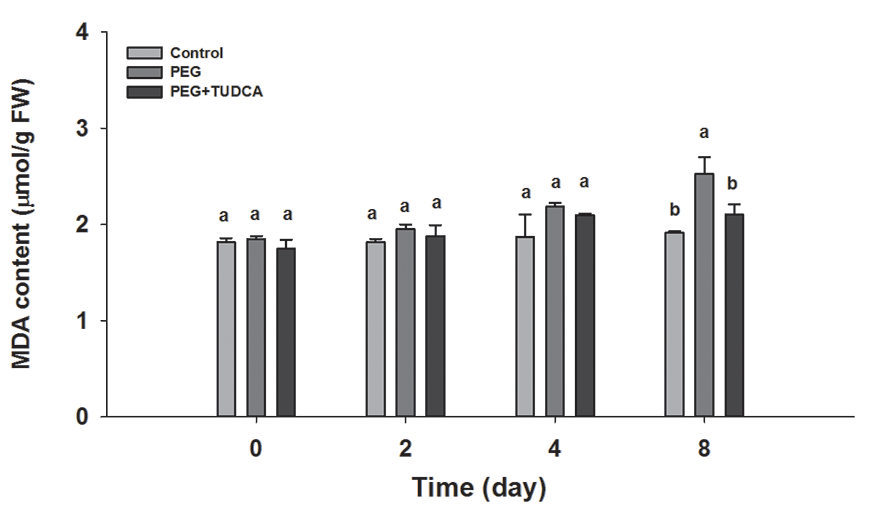

Supplement: Figure S5 — Time course of MDA content in wheat leaves under different treatments. MDA content in wheat leaves of control, PEG, and PEG + TUDCA (100 μg·mL−1) treatment groups after 0, 2, 4, and 8 days of PEG stress. Data are shown as mean ± SD (n = 4). Different letters (a or b) indicate significant difference between the groups (P < 0.05). [file Image5.TIF]

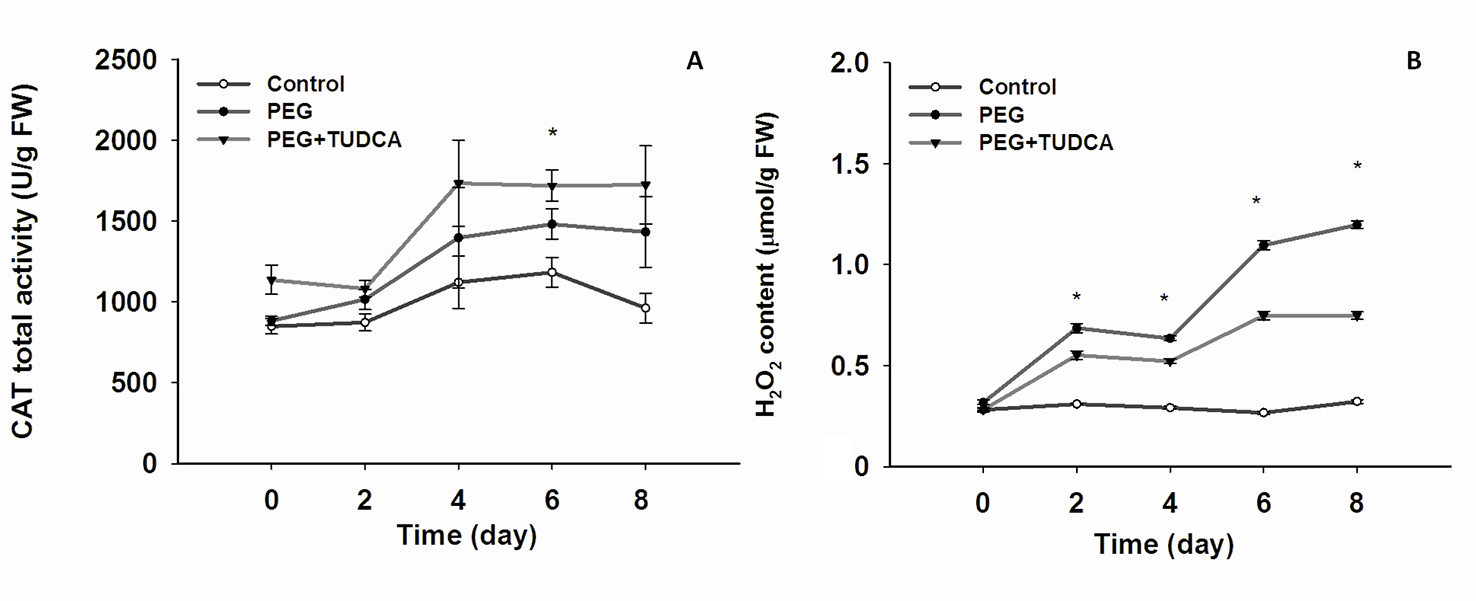

Supplement: Figure S6 — Time course response of the CAT activity and H2O2 content in wheat leaves subjected to different treatments. (A) CAT activity in wheat leaves of control, PEG, and PEG ± TUDCA treatment groups under PEG stress for 0, 2, 4, 6, and 8 days; (B) H2O2 content in wheat leaves of control, PEG, and PEG + TUDCA treatment groups under PEG stress for 0, 2, 4, 6, and 8 days. Data are shown as mean ± SD (n = 4) of three independent experiments. Significant difference between the groups (P < 0.05) is indicated by an *. [file Image6.TIF]

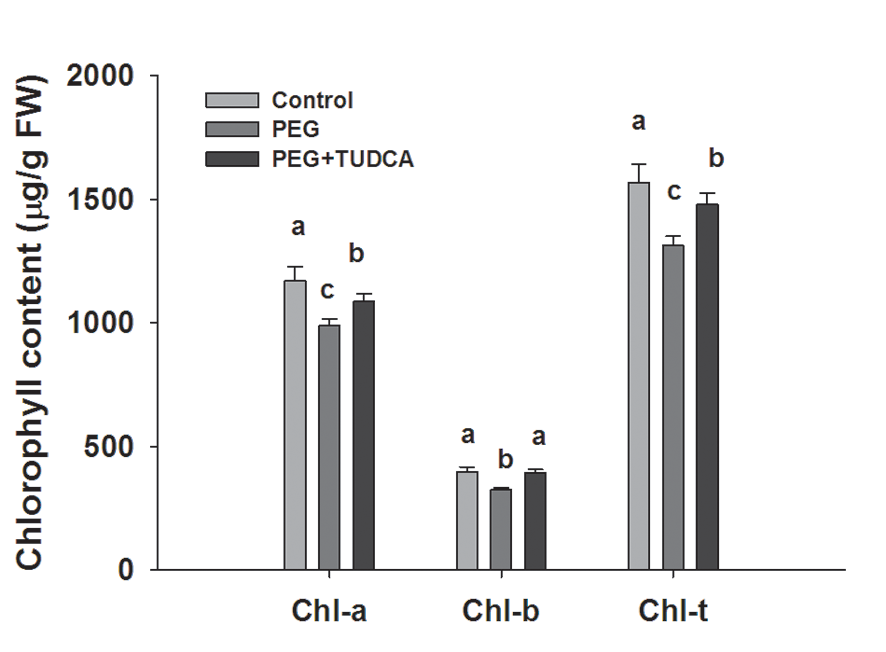

Supplement: Figure S7 — Chlorophyll content changes in wheat seedlings under different treatments. Chlorophyll a, chlorophyll b and chlorophyll total content in wheat leaves of control, PEG, and PEG + TUDCA treatment groups under PEG stress for 4 days. Chl-a, chlorophyll a; Chl-b, chlorophyll b; Chl-t, total chlorophyll. Data are shown as mean ± SD (n = 4). Different letters (a, b or c) indicate significant difference between the groups (P < 0.05). [file Image7.TIF]

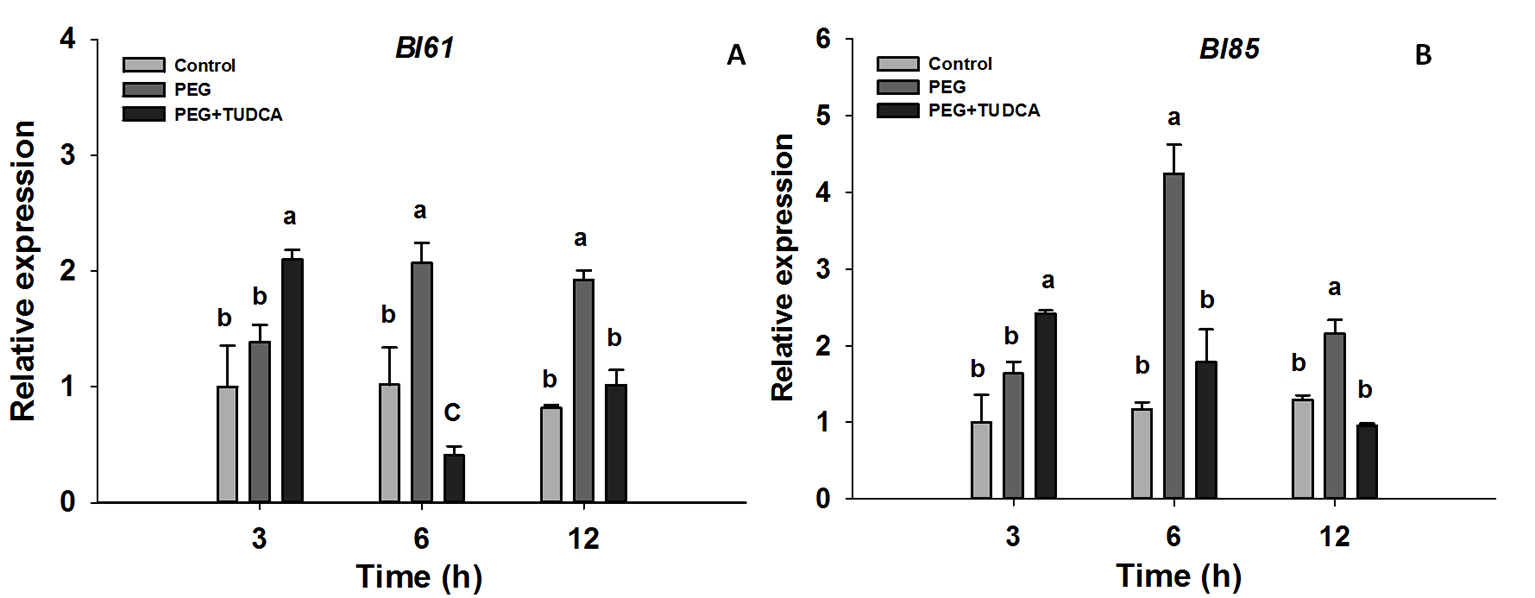

Supplement: Figure S8 — Time course of BI61 and BI85 expression in wheat leaves. (A) The relative expression of BI61 of control, PEG, and PEG + TUDCA treatment groups after 3, 6, and 12 h of PEG stress; (B) The relative expression of BI61 of control, PEG, and PEG + TUDCA treatment groups after 3, 6, and 12 h of PEG stress. Data are shown as mean ± SD (n = 3) of three independent experiments. Different letters (a or b) indicate significant difference between the groups (P < 0.05). [file Image8.TIF]
