# Supplementary material for: Osmotic Stress Induced Cell Death in Wheat Is Alleviated by Tauroursodeoxycholic Acid and Involves Endoplasmic Reticulum Stress–Related Gene Expression
Source: Front Plant Sci. 2017 May 3;8:667. doi: 10.3389/fpls.2017.00667 (PMC5413500; doi:10.3389/fpls.2017.00667)
Supplement: Supplementary file 9 [file Table1.DOCX]

**Supplementary data**

Table S1 qPCR primer sequences of endoplasmic reticulum associated genes

| **Number** | **Name** | **qPCR primer sequences** |
| --- | --- | --- |
| 1 | TabZIP60BF | GTTCTTGCCTTGTTCAGTCTGT |
|  | TabZIP60BR | CTTTTGCCTCTCTAGGATCTCC |
| 2 | TabZIP60DF | CTTATTCTCTGTTCTTGCCTTG |
|  | TabZIP60DR | CCTCATTCTTTTGCCTCTCTA |
| 3 | TaBiP1F | GCTATTGCCTATGGTTTGGA |
|  | TaBiP1R | CCTTGCCGTGCTTCTTCT |
| 4 | TaBiP2F | GTCAAGCGTCTCATTGGAAG |
|  | TaBiP2R | GGTATGCCTCAGCGGTCT |
| 5 | TaPDIL8-1F | GCTGGTTACGCTGAGATACG |
|  | TaPDIL8-1R | GATGAAGTGGGAGAATGATTTG |
| 6 | TaGRP94F | ATGATGAAGAGAAGAAGCCAAAGAC |
|  | TaGRP94R | GGGGGAACAAAGAGCAAAGC |
| 7 | TaBI61F | GCTGGTTACGCTGAGATACG |
|  | TaBI61R | GATGAAGTGGGAGAATGATTTG |
| 8 | TaBI85F | ATGATGAAGAGAAGAAGCCAAAGAC |
|  | TaBI85R | GGGGGAACAAAGAGCAAAGC |
